# Safe and effective treatment of venous Thromboembolism associated with Cancer: focus on direct Oral Anticoagulants in Asian patients

**DOI:** 10.1186/s40164-022-00331-9

**Published:** 2022-10-27

**Authors:** Lai Heng Lee, Pongwut Danchaivijitr, Noppacharn Uaprasert, Harinder Gill, Dennis Lee Sacdalan, Gwo Fuang Ho, Rajiv Parakh, Paresh Pai, Jen-Kuang Lee, Nannette Rey, Alexander T. Cohen

**Affiliations:** 1 Haematology Department, Singapore General Hospital, Bukit Merah, Singapore; 2 Division of Medical Oncology, Department of Internal Medicine, Siriraj Hospital, Mahidol University, Bangkok, Thailand; 3 Research Unit in Translational Hematology, Division of Hematology, Department of Medicine, Faculty of Medicine, Chulalongkorn University, Bangkok, Thailand; 4 Department of Medicine, The University of Hong Kong, 102 Pokfulam Road, Pokfulam Hong Kong, Pokfulam, Hong Kong China; 5 College of Medicine, University of the Philippines, Manila, Philippines; 6 Clinical Oncology, University Malaya Medical Centre, Kuala Lumpur, Malaysia; 7 Division of Peripheral Vascular & Endovascular Service, Medanta–Medicity, Gurgaon, Haryana India; 8 Vascular and Endovascular Surgery, The Vascular Clinic, Mumbai, India; 9 Cardiology Department, National Taiwan University Hospital, Taipei, Taiwan; 10 de La Salle Medical and Health Sciences Institute, Dasmarinas Cavite, Philippines; 11 Department of Haematological Medicine, Guy’s and St Thomas’ Hospitals, NHS Trust, King’s College, London, UK

**Keywords:** Anticoagulation, Cancer, Venous Thromboembolism

## Abstract

Cancer-associated thrombosis (CAT) poses a significant disease burden and the incidence in Asian populations is increasing. Anticoagulation is the cornerstone of treatment, but can be challenging due to the high bleeding risk in some cancers and the high risk of recurrent venous thromboembolism (VTE) in patients with malignancies. Direct oral anticoagulants (DOACs) are well established as first-choice treatments for VTE in non-cancer patients, offering a more convenient and less invasive treatment option than low-molecular-weight heparin (LMWH). Asian patients have exhibited comparable efficacy and safety outcomes with other races in trials of DOACs for VTE in the general population. Although no specific data are available in Asian patients with CAT, results from randomized controlled trials of apixaban, edoxaban, or rivaroxaban versus the LMWH, dalteparin, indicate that DOACs are a reasonable alternative to LMWH for anticoagulation in Asian patients with CAT. This is further supported by analyses of real-world data in Asian populations demonstrating the efficacy and safety of DOACs in Asian patients with CAT. Apixaban, edoxaban, or rivaroxaban are recommended in the most recently updated international guidelines as first-line therapy for CAT in patients without gastrointestinal or genitourinary cancers and at low risk of bleeding. An increased risk of major gastrointestinal bleeding was evident with edoxaban or rivaroxaban, but not apixaban, versus dalteparin in the clinical trials, suggesting that apixaban could be a safe alternative to LMWH in patients with gastrointestinal malignancies. Determining the optimal anticoagulant therapy for patients with CAT requires careful consideration of bleeding risk, tumor type, renal function, drug–drug interactions, financial costs, and patients’ needs and preferences.

## Introduction

Thrombosis is a common and detrimental complication of cancer contributing to significant morbidity and mortality worldwide [1–4], with venous thromboembolism (VTE) as the second-leading cause of mortality in cancer patients [5, 6]. Among Asian populations, 16–40% of VTE cases are associated with malignancies [7–9].

Cancer-associated thrombosis (CAT) poses a significant disease burden. The incidence of first VTE was an alarming 54-fold higher (5,800 vs. 107 per 100,000 person-years) in patients with active cancer compared to those without cancer in a UK population-based study [4]. While the incidence of VTE is reportedly lower in Asians than in Caucasians, the annual rate of VTE in Asia is rising, with cancer identified as one of the most common risk factors [10, 11]. A population-based study in Taiwan found an estimated incidence of VTE of 9.9 per 1,000 person-years among patients with newly diagnosed cancer [12]. Other population-based studies in Asia have reported significant increases in CAT over recent decades [13, 14]. The rising incidence of CAT may be partly explained by an increasing number of elderly patients undergoing cancer treatments, a greater awareness of CAT, and increased detection of incidental VTE [3, 15].

Consistent with the hypercoagulable state that is known to be induced by malignancy [2, 16, 17], rates of VTE in newly diagnosed cancer patients in Asian countries, ranging from 2.2 to 11.5 times higher than those in the general population, have been reported [18]. Risk factors contributing to the development of VTE in patients with cancer and accounting for the substantial variation in VTE risk observed among this group include tumor-related factors (cancer type, stage, tumor-derived factors, metastases), patient-related factors (genetics, age, weight, VTE history, immobility), cancer treatment-related factors (chemotherapy, hormone therapy, radiotherapy, surgery, central venous catheter use), and biomarkers (platelet count, leucocyte count, prothrombotic variants, and natural anticoagulant deficiencies) [16, 19–22].

Adequate anticoagulation remains the cornerstone treatment for CAT. However, optimizing anticoagulant therapy for patients with CAT is challenging due to factors such as the higher bleeding risk in certain cancer types, particularly gastrointestinal (GI) and genitourinary (GU) cancers with intact primary tumors, potential multiple drug interactions, chemotherapy-induced thrombocytopenia and altered GI absorption of oral anticoagulants due to vomiting, diarrhea, or mucositis [1, 23, 24].

CAT carries a high risk of recurrence despite anticoagulant therapy [1]. Following an index VTE, patients with malignancy have a 3-fold higher risk of VTE recurrence than patients without cancer, even when receiving anticoagulation [25]. An American population-based study following incident VTE patients for > 10 years revealed that those with active cancer had higher VTE recurrence rates than those with idiopathic VTE or secondary non-cancer-associated VTE (43.4% vs. 27.3% and 18.1%, respectively), suggesting the need for long-term anticoagulation in this group [26]. Data on VTE recurrence in Asian patients with cancer are limited, but a Korean study on CAT in patients with advanced solid cancers showed the 6-month and 12-month cumulative incidences of recurrent VTE were 20.6% and 27.0%, respectively, of which more than 50% recurred as pulmonary embolism [27]. Patients with recurrent VTE had significantly shorter overall survival than those without (median 8.4 months vs. 13.0 months, *p =* 0.001).

There is a well-recognized need for better treatment options for VTE in Asian patients [10].Encouragingly, Asian patients have exhibited comparable efficacy and safety outcomes with other races in trials of direct oral anticoagulants (DOACs) for VTE in the general population [10]. There is, however, limited high quality data on the efficacy and safety of DOACs in Asian patients with CAT [28]. Here we review the evidence for the treatment of CAT from an Asian perspective and discuss the potential impact of newly published data on treatment guidelines and the standard of care.

## Diagnosis and treatment of CAT in Asia

The rates of VTE are lower in Asia compared to the Western population, but 16–40% of Asian VTE cases are cancer-associated (particularly with advanced-stage cancer), making malignancy the most common acquired risk for VTE in Asians [29, 30]. In general, diagnostic assessments involve clinical assessment, plasma d-dimer measurement and imaging studies. The prediction model developed by Khorana et al. [31] has been recommended as an assessment tool for CAT by the National Comprehensive Cancer Network (NCCN) and the Chinese Society of Clinical Oncology (CSCO)[32].

Until recently, low-molecular-weight heparins (LMWHs) have been the standard of care for CAT in both the West and Asia. This treatment is based on the results of the CLOT trial comparing the LMWH dalteparin with vitamin K antagonist (VKA) therapy [33], and as a consequence of the difficulties associated with long-term adherence to VKAs, especially in patients with malignancy [16, 28]. Moreover, the warfarin dose required to achieve an international normalized ratio (INR) of 2 or 3 is often lower in Asian patients due to pharmacogenetic differences in warfarin metabolism involving *VKORC* genes with increased prevalence in Asian populations [34]. Asian patients are therefore at increased risk of elevated INR, which further limits the acceptability of VKA therapy in this population. Adherence to long-term treatment with LMWHs is also poor [1, 23, 35–37]. Reasons for withdrawal of LMWH include injection site pain and bruising, bleeding, the inconvenience of daily subcutaneous injections, and cost [23, 37, 38].

DOACs are established as first-choice treatments for VTE in non-cancer patients and offer a more convenient and less invasive treatment option than LMWH [39, 40]. DOACs, including the factor Xa inhibitors rivaroxaban, edoxaban, and apixaban, and the direct thrombin inhibitor dabigatran, are administered orally in fixed doses, unlike LMWHs which are administered via subcutaneous injection and unlike warfarin which requires adjustment and laboratory monitoring [1, 40].

Recent studies suggest that DOACs may be as effective and safe as conventional therapeutic approaches for CAT. Preliminary evidence supporting the use of DOACs in the treatment of CAT came from pooled analyses of the subgroup of patients with cancer in the phase 3 DOAC studies conducted in the general population [41–47]. A meta-analysis of the pooled data revealed that DOACs (*n =* 595) were as effective and as safe as conventional VKA treatment (*n =* 537) for the prevention of recurrent VTE in patients with cancer, with nearly a 40% reduction in VTE recurrence [48]. The proportion of Asian patients included in these studies is shown in Table1, and subgroup analyses have shown Asian patients had at least comparable efficacy and safety to other races [10, 28]. Notably, data from the trials suggest that no weight-based DOAC dose adjustment is necessary [10], which is particularly relevant for Asian patients who tend to have lower body mass indices than non-Asians. These studies were, however, not specifically designed to test the efficacy and safety of DOACs in patients with cancer and may not accurately reflect the target population of patients with cancer, particularly as those with active cancer were often excluded from the trials [23]. The studies’ use of VKAs as the comparator rather than the LMWH standard of care for CAT further limits the relevance of the results for patients with cancer [30].

**Table 1:** Proportion of Asian patients enrolled in randomized controlled trials of DOACs

Study	Treatment	Total population N	Asians N (%)
RCTs for treatment of VTE			
RE-COVER (41)	Dabigatran vs. standard care	2539	65 (2.6%)
RE-COVER II (43)	Dabigatran vs. standard care	2589	537 (20.7%)
EINSTEIN-DVT (44)	Rivaroxaban vs. standard care	3449	494 (14.3%)
EINSTEIN-PE (45)	Rivaroxaban vs. standard care	4832	287 (5.9%)
AMPLIFY (46)	Apixaban vs. standard care	5395	NR
Hokusai-VTE (47)	Edoxaban vs. standard care	8240	1727 (21.0%)
RCTs for treatment of cancer-associated VTE			
Hokusai-VTE Cancer (50)	Edoxaban vs. standard care	1046	NR
SELECT-D (51)	Rivaroxaban vs. standard care	406	< 15 (< 4%)^a^
CARAVAGGIO (52)	Apixaban vs. edoxaban	1155	NR
ADAM VTE (53)	Apixaban vs. standard care	300	2 (0.7%)

NR, not reported; RCT, randomized controlled trial

^a^Data extrapolated from report of number of patients with White ethnicity

Evidence of the efficacy of DOACs versus dalteparin in selected patients with active cancer has since been demonstrated in four key randomized controlled trials (RCTs): the Hokusai VTE Cancer study, the SELECT-D trial, the ADAM VTE trial and the CARAVAGGIO trial (Table2) [49–52]. Asian patients were rarely included in these cancer-specific RCTs (Table1), so no Asian-specific CAT data is available, but together with the best available clinical evidence for the use of DOACs in general populations of Asian patients with VTE, the results indicate that DOACs are a reasonable alternative to LMWH for Asian patients with CAT [28].

**Table 2:** Outcomes of randomized trials comparing DOACs and dalteparin in the treatment of CAT

	CARAVAGGIO (52)			ADAM-VTE (53)		Hokusai VTE Cancer (50)		SELECT-D (51)	
	Apixaban (*n=*576)		Dalteparin (*n=*579)	Apixaban (*n=*150)^a^	Dalteparin (*n=*150)^b^	Edoxaban (*n=*522)	Dalteparin (*n=*524)	Rivaroxaban (*n=*203)	Dalteparin (*n*=203)
Recurrent VTE, n (%)	32 (5.6)		46 (7.9)	1 (0.7)	9 (6.3)	41 (7.9)	59 (11.3)	8 (4.0)^d^	18 (11.0)^d^
HR (95% CI)^c^	0.63 (0.37–1.07)			0.099 (0.013–0.78)		0.71 (0.48–1.06)		0.43 (0.19–0.99)	
Major bleeding, n (%)	22 (3.8)		23 (4.0)	0	2 (1.4)	36 (6.9)	21 (4.0)	11 (6.0)^d^	6 (4.0)^d^
HR (95% CI)^c^	0.82 (0.40–1.69)			0.0 (0.0–)		1.77 (1.03–3.04)		1.83 (3–11)	
Major GI bleeding, n (%)	11 (1.9)		10 (1.7)	0	0	20 (3.8)	6 (1.1)	8 (3.9)	4 (2.0)
HR (95% CI)^c^	1.05 (0.44–2.50)			NE		NR		NR	
CRNM bleeding, n (%)	52 (9.0)	35 (6.0)		9 (6.2)	7 (4.2)	76 (14.6)	58 (11.1)	25 (13.0)^d^	7 (4.0)^d^
HR (95% CI)^c^	1.42 (0.88–2.30)			NR		1.38 (0.98–1.94)		3.76 (1.63–8.69)	
Mortality, n (%)	135 (23.4)	153 (26.4)		23 (16)	15 (11)	206 (39.5)	192 (36.6)	48 (23.6)	56 (27.6)
HR (95% CI)^c^	0.82 (0.62–1.09)			1.40 (0.82–2.43)		1.12 (0.92–1.37)		NR	

CAT, cancer-associated thrombosis; CI, confidence interval; CRNM, clinically relevant non-major; DOAC, direct oral anticoagulant; DVT, deep vein thrombosis; GI, gastrointestinal; HR, hazard ratio; NE, not evaluated; NR, not reported; PE, pulmonary embolism; PET, positron emission tomography; SD, standard deviation; VTE, venous thromboembolism

^a^Primary analysis population (***n =*** 145)

^b^Primary analysis population (***n =*** 142)

^c^DOAC versus dalteparin

^d^Cumulative percentages

## Major studies evaluating DOACs for CAT – relevance to the Asian Population

### The Hokusai VTE Cancer study

The Hokusai VTE Cancer study was the first to compare a DOAC (edoxaban) with an LMWH (dalteparin) for the treatment of CAT. The multicenter, randomized, open-label study demonstrated that once-daily oral edoxaban (median duration 211 days) was non-inferior to once-daily subcutaneous dalteparin (median duration 184 days) for the combined outcome of recurrent thrombosis and major bleeding (hazard ratio [HR] 0.97; 95% confidence interval [CI] 0.70–1.36, *p =* 0.006 for non-inferiority) [49]. However, in this study, major bleeding (a secondary endpoint) was experienced by significantly more edoxaban than dalteparin recipients (6.9% vs. 4.0%, HR 1.77; 95% CI 1.03–3.04, *p =* 0.04); this increase in bleeding was mainly due to upper GI bleeding in patients with cancer of the upper GI tract [49].

A post hoc subgroup analysis showed that there was a significant increase in major bleeding with edoxaban in patients with GI cancers (12.7% vs. 3.6% with dalteparin; HR, 4.0; 95% CI, 1.5–1.06; *p =* 0.005) [53]. In patients with GI cancer, upper GI bleeding accounted for most (76.2%) of the 21 major bleeding events in the edoxaban group, and none of 5 major bleeding events in the dalteparin group. Three quarters of the upper GI bleeding events in patients with GI cancer occurred in patients with unresected tumors, suggesting that patients with intact GI tumors had the greatest risk of bleeding with edoxaban.

### The SELECT-D trial

The multicenter, randomized, open-label, pilot SELECT-D (Anticoagulation Therapy in Selected Cancer Patients at Risk of Recurrence of Venous Thromboembolism) trial compared rivaroxaban (15mg orally twice daily for 3 weeks, then 20mg once daily for 6 months; *n =* 203) with subcutaneous dalteparin (200 IU/kg daily during month 1, then 150 IU/kg daily for months 2–6; *n =* 203) [50]. Rivaroxaban was associated with a lower risk of cumulative VTE recurrence at 6 months (primary outcome) compared with dalteparin (4.0% vs. 11.0%; HR 0.43; 95% CI 0.19–0.99) [50]. However, for the secondary outcomes of major bleeding and clinically relevant non-major bleeding (CRNMB), rivaroxaban was associated with significantly higher rates than dalteparin; 6-month cumulative major bleed and cumulative CRNMB rates of 6% versus 4% (HR 1.83; 95% CI 0.68–4.96) and 13% versus 4% (HR 3.76; 95% CI 1.63–8.69), respectively [50]. CRNMB events were mostly GI or GU and most major bleeding events were GI, but there were no central nervous system bleeds [50]. Patients with esophageal or gastroesophageal cancer tended to have more major bleeds with rivaroxaban (36%) than with dalteparin (11%). Recruitment of patients with cancer of the esophagus or gastroesophageal junction into the study was stopped when the increased risk of major bleeding with rivaroxaban in patients with upper GI tumors became clear. The increased risk of bleeding appears to be an essential problem in mucosal types of cancer [54].

### The CARAVAGGIO trial

In the multinational, randomized, investigator-initiated, open-label, non-inferiority CARAVAGGIO trial, patients with cancer and VTE received either oral apixaban (10mg twice daily for 7 days followed by 5mg twice daily; *n =* 576) or subcutaneous dalteparin (200 IU/kg once daily for 1 month followed by 150 IU/kg daily; *n =* 579) for 6 months [51]. Approximately one-third of the patients had GI cancer. Objectively confirmed recurrent VTE during treatment (primary outcome) occurred at similar rates in each group; 5.6% with apixaban versus 7.9% with dalteparin (HR 0.63; 95% CI 0.37–1.07; *p <* 0.001 for non-inferiority; *p =* 0.09 for superiority) [51]. Major bleeding (principal safety outcome) occurred at similar rates in apixaban and dalteparin recipients (3.8% vs. 4.0%; HR 0.82; 95% CI 0.40–1.69; *p =* 0.60) [51]. Major GI bleeding rates were also similar between the two groups (1.9% vs. 1.7%, HR 1.05; 95% CI 0.44–2.50) [51]. Analysis of event-free survival in this study (composite of recurrent VTE, major bleeding, or death) showed significantly higher rates with apixaban than with dalteparin (73.3% vs. 68.6%; HR 1.36; 95% CI 1.05–1.76). The similar rates of major bleeding observed between apixaban and dalteparin in the CARAVAGGIO trial are in contrast to those previously seen with other DOACs (rivaroxaban [SELECT-D trial]; edoxaban [Hokusai VTE Cancer study]), where DOACs exhibited significantly higher rates of major bleeding compared with dalteparin [47, 50, 51].

A subanalysis of the CARAVAGGIO trial has shown that rates of major GI bleeding in patients with GI cancer were low and similar between the two treatment groups (lower GI bleeding occurred in 3 of 188 patients with GI cancer in the apixaban treatment group and 3 of 187 patients in the dalteparin group, and upper GI bleeding occurred in 4 and 3 patients, respectively) [55]. All major GI bleeding events reported with apixaban in patients with GI cancer occurred in those with unresected tumors (5 of 121 patients with unresected colorectal cancer, 2 of 44 patients with unresected pancreatic or hepatobiliary cancer, and 2 of 18 patients with unresected upper GI cancer) [55].

### The ADAM VTE trial

The multicenter, randomized, open-label ADAM VTE trial comparing apixaban to dalteparin in CAT found similar findings to the CARAVAGGIO trial, with major bleeding (primary outcome) occurring in a statistically similar number of patients during approximately 6 months of treatment (0% of 145 patients receiving apixaban and 1.4% of 142 dalteparin recipients, *p =* 0.138) [52]. There were significantly fewer recurrent VTE events (secondary outcome) in apixaban recipients than dalteparin recipients (0.7% vs. 6.3%; HR 0.099; 95% CI 0.013–0.780, *p =* 0.0281). Furthermore, apixaban was associated with better quality of life (excess bruising, stress, irritation and worry, burden of delivery, and overall satisfaction with anticoagulant therapy) than dalteparin.

### Meta-analyses

Several meta-analyses of the four key DOAC trials in cancer patients (Hokusai VTE Cancer, SELECT-D, CARAVAGGIO, and ADAM VTE) have been conducted (Table3) [35, 39, 56–60]. Each had similar findings in terms of efficacy, despite employing different statistical methods, with the factor Xa inhibitor DOACs reducing the risk of recurrent VTE compared with dalteparin. A low level of heterogeneity and the consistency of efficacy results between the studies reflect the generalizability of the improved efficacy of oral factor Xa inhibitors versus dalteparin, indicating that further studies are unlikely to change this finding [39, 56, 57]. In contrast, between-study heterogeneity was apparent for outcomes of major bleeding, with the ADAM VTE and CARAVAGGIO trials showing no increase in overall major bleeding risk with apixaban versus dalteparin [39, 51, 52]. Excluding both apixaban studies, a meta-analysis of Hokusai VTE Cancer and SELECT-D showed edoxaban and rivaroxaban to have a significantly increased risk of major bleeding compared with dalteparin [35]. Similarly, excluding only the CARAVAGGIO trial, a meta-analysis of Hokusai VTE Cancer, SELECT-D, and ADAM VTE showed the DOACs to be associated with a significant increase in major bleeding compared with dalteparin [56]. Notably, compared with Hokusai VTE Cancer, SELECT-D, and ADAM VTE, the CARAVAGGIO trial included a smaller proportion of patients with upper GI cancers who are more susceptible to major bleeding [61]. Considerations such as these introduce uncertainty as to whether the differences in safety profiles are agent-, regimen- or trial-specific [39, 57]. Without head-to-head comparisons it is difficult to conclusively determine if one DOAC is safer than another [61]. In a recent meta-analysis that identified 6 studies comprising 3,542 Asian and 23,481 non-Asian patients, DOAC significantly reduced major and non-major bleeding in Asian patients compared to vitamin K antagonist [62].

**Table 3:** Outcomes of meta-analyses of RCTs of DOACs versus LMWH for the treatment of CAT

Meta-analysis	Number of patients analyzed	RR or HR for DOACs versus LMWH (95% CI)		
		**VTE recurrence**	**Major bleeding**	**CNRMB**
Mulder, et al.^a^ (59)	2607	0.68 (0.39–1.17)	1.36 (0.55–3.35)	1.63 (0.73–3.64)
Giustozzi et al.^b^ (39)	2894	0.62 (0.43–0.91)	1.31 (0.83–2.08)	1.51 (1.09–2.09)
Moik, et al.^b^ (58)	2894	0.62 (0.43–0.91)	1.31 (0.83–2.08)	1.65 (1.19–2.28)
Tao, et al.^b^ (61)	2894	0.62 (0.43–0.91)	1.31, (0.83–2.08)	1.65 (1.19–2.28)
Haykal, et al.^b^ (57)	2907	0.62 (0.44–0.87)	1.33 (0.45–4.22)	1.58 (1.11–2.24)
Saleem, et al.^b^ (60)	2907	0.54 (0.23–1.28)	1.38 (0.45–4.22)	1.77 (0.49–6.40)

Abbreviations: CAT, cancer-associated thrombosis; CI, confidence interval; DOAC, direct oral anticoagulant; CRNMB, clinically relevant non-major bleeding; HR, hazard ratio; LMWH, low molecular weight heparin; RCT, randomized controlled trial; RR, risk ratio; VTE, venous thromboembolism

^a^Hokusai VTE cancer, SELECT-D and Caravaggio

^b^Hokusai VTE cancer, SELECT-D, ADAM VTE and Caravaggio

### Real-world evidence

Supplementing the results of RCTs, analyses of real-world data of the efficacy and safety of DOACs have been conducted in US and Asian populations of patients with CAT [18, 63–66]. These studies show that DOACs are effective and safe alternatives to LMWH for the treatment of CAT in real-world practice.

A real-world analysis of US medical and pharmacy claims data in newly diagnosed patients with CAT found a significantly lower risk of recurrent VTE in patients prescribed rivaroxaban (*n =* 707) compared with those prescribed LMWH (*n =* 660) or warfarin (*n =* 1061) [65]. There was a non-significant trend for lower VTE rates with rivaroxaban compared to LMWH recipients (13.2% vs. 17.1%; *p =* 0.060) at 6 months, which became significant at 12 months with rivaroxaban recipients exhibiting a 28% decreased risk of VTE recurrence (16.5% vs. 22.2%; HR 0.72; 95% CI 0.52–0.95; *p =* 0.024). VTE recurrence rates were significantly lower for rivaroxaban versus warfarin at 6 months (13.2% vs. 17.5%; *p =* 0.014) and 12 months (15.7% vs. 19.9%; *p =* 0.017), with a 26% decreased risk of VTE recurrence (HR 0.74; 95% CI 0.56–0.96; *p =* 0.028). Rates of major bleeding at 6 months were similar for rivaroxaban versus LMWH (8.2% vs. 8.3%) and also for rivaroxaban versus warfarin (9.0% vs. 8.7%) with no significant differences between treatment groups. Notably, the majority of major bleeding events were GI in each treatment group.

Complementing the above findings, but with a focus on apixaban, an analysis of pooled data from four US claims databases in patients with VTE and active cancer found a lower risk of VTE in patients prescribed apixaban (*n =* 3393) compared with those prescribed LMWH (*n =* 6108) or warfarin (*n =* 4585) [64]. At 6 months, an approximately 30–40% lower risk of recurrent VTE was observed with apixaban versus LMWH (HR 0.61; 95% CI 0.47–0.81) and versus warfarin (HR 0.68; 95% CI 0.52–0.90). Furthermore, apixaban had a 37% reduced risk of major bleeding compared with LMWH (HR 0.63; 95% CI 0.47–0.86) and a similar risk of major bleeding compared with warfarin (HR 0.73; 95% CI 0.53–1.00).

Providing a direct comparison between apixaban and rivaroxaban, 750 patients with CAT treated with apixaban (*n =* 224), rivaroxaban (*n =* 163), or enoxaparin (*n =* 363) were followed prospectively in a clinical setting in the US [66]. Recurrence of VTE was similar across treatment groups with HRs of 1.31 (95% CI 0.51–3.36) for apixaban versus rivaroxaban, 1.14 (95% CI 0.54–2.42) for apixaban versus enoxaparin, and 0.85 (95% CI 0.36–2.06) for rivaroxaban versus enoxaparin. Rates of major bleeding were also similar across treatment groups with HRs of 0.73 (95% CI 0.32–1.66), 0.89 (95% CI 0.43–1.84), and 1.23 (95% CI 0.61–2.50), respectively. Rivaroxaban was associated with a significantly higher rate of CRNMB compared with apixaban (8.8% vs. 0.6%; *p =* 0.03) and enoxaparin (8.8% vs. 2.2%; *p =* 0.01).

Presenting an Asian perspective, a retrospective analysis of 1109 patients with CAT identified from a medical records database in Taiwan found the use of DOACs (374 rivaroxaban; 51 apixaban, 35 edoxaban, and 11 dabigatran recipients) to be associated with similar risks for recurrent VTE and major bleeding as use of LMWH (508 enoxaparin recipients) at 12 months of follow-up [18]. The composite rate of recurrent VTE or major bleeding events was 14.1% in DOAC recipients versus 17.4% in enoxaparin recipients (weighted HR 0.77; 95% CI 0.56–1.07; *p =* 0.11). In contrast to the increased risk of major GI bleeding observed with edoxaban and rivaroxaban in the Hokusai VTE cancer and SELECT-D RCTs [49, 50], the rate of GI bleeding was significantly lower in the DOAC group than in the LMWH group (HR 0.29; 95% CI 0.15–0.59; *p <* 0.001) [18]. There were no differences in major bleeding or major GI bleeding between patients with GI tract cancer and those with non-GI tract cancer.

With regard to recurrent VTE and bleeding, similar findings were reported in a Korean retrospective cohort study involving patients with CAT treated with DOACs (*n =* 132), LMWHs (*n =* 119), or other anticoagulants (*n =* 372) for ≥ 3 months [63]. During the initial 6 months of treatment, the respective overall cumulative incidence of VTE recurrence and bleeding events were 16.7% and 12.3% with DOACs, 8.3% and 11.0% with LMWH, and 20.7% and 30.7% with other anticoagulants, with no significant differences between DOACs and LMWH. A subgroup analysis for patients treated with DOACs showed that there were no differences in VTE recurrence or bleeding rates in patients with upper GI tract cancer versus other cancers.

## International guidelines for the treatment of CAT

Recently updated guidelines from major societies recognize that DOACs have a role in treating CAT, but may not be suitable for all patients due to their potential to cause bleeding [67]. Recommendations from current international treatment guidelines for the treatment of CAT differ with respect to which DOACs they recommend (Table4) and these are largely influenced by the availability of data from key RCTs during preparation of the guidelines [68–75]. The 2021 American College of Chest Physicians (ACCP), 2022 National Comprehensive Cancer Network (NCCN) and 2021 American Society of Hematology (ASH) guidelines, all of which were updated post-CARAVAGGIO, support use of anti-factor Xa DOACs for the treatment of CAT [73–75]. Guidelines from the American College of Chest Physicians (ACCP) strongly recommend treatment with apixaban, edoxaban or rivaroxaban over LMWH, but remark that apixaban or LMWH may be the preferred option in patients with luminal GI malignancies [75]. NCCN guidelines specify preference for apixaban, edoxaban, or rivaroxaban over LMWH in patients without gastric or gastroesophageal lesions, but acknowledge that apixaban may be safer than edoxaban or rivaroxaban in patients with such lesions [74]. Caution is advised in patients with GU cancer [74]. ASH guidelines recommend initial treatment using LMWH, rivaroxaban, or apixaban and short- to long-term anticoagulation with apixaban, edoxaban, or rivaroxaban, but advise caution in patients with GI cancer [73]. National Institute for Health and Care Excellence (NICE) guidelines recommend DOACs for patients with active cancer and confirmed proximal VTE [72]. The most recent guidelines from the American Society of Oncology (ASCO), the International Society on Thrombosis and Haemostasis (ISTH) and the International Initiative on Thrombosis and Cancer (ITAC) were not in a position to consider the CARAVAGGIO or ADAM-VTE results and specifically recommend edoxaban or rivaroxaban for patients with non-GI cancers at low bleeding risk; otherwise LMWH is recommended as the preferred anticoagulant [68, 70, 71]. Guidelines from the European Society of Cardiology (ESC) suggest that edoxaban or rivaroxaban be considered as an alternative to LMWH, but that use in patients with GI cancer should be undertaken with caution [69].

**Table 4:** Current international treatment guideline recommendations for the treatment of cancer-associated VTE

Guideline		Recommendations		
		**Initial treatment**	**Treatment duration**	
ISTH 2018^a^ (69)		• Patients with low bleeding risk and no drug–drug interactions: edoxaban or rivaroxaban; LMWHs are acceptable alternatives. • Patients with high bleeding risk^c^: LMWH; edoxaban or rivaroxaban as an alternative if no potential DDI.	• No specific recommendation.	
ESC 2019^a^ (70)		• PE and cancer: LMWH for the first 3–6months (classIIa, levelA). • Edoxaban (classIIa, level B) or rivaroxaban (classIIa, levelC) may be used except in GI cancer patients.	• Extend indefinitely or until the cancer is cured (classIIa, levelB). • Consider LMWH, NOAC or VKA.	
ASCO 2019^a^ (71)		• LMWH, UFH, fondaparinux or rivaroxaban.	• Offer LMWH, NOACs or VKAs beyond the initial 6months to select patients with active cancer, such as those with metastatic disease or those receiving chemotherapy. o LMWH, edoxaban or rivaroxaban preferred. o LMWH preferred in settings with increased bleeding risk. • Assess intermittently to ensure a continued favorable risk-benefit profile	
ITAC 2019^a^ (72)		• LMWH when CrCl ≥ 30mL/min (grade1B). • Rivaroxaban (first 10days) or edoxaban (started after initial LMWH/UFH for 5 days) can be used for initial treatment if CrCl ≥ 30mL/min and patient is not at high risk of GI or GU bleeding (grade1B).	• LMWH or DOACs for ≥ 6 months (grade1A) – DOACs when CrCl ≥ 30mL/min if no impairment in GI absorption or strong DDIs (grade1A), but caution advised in GI malignancies, especially upper GI tract. • After 6months, termination or continuation of anticoagulation based on benefit-risk ratio, tolerability, drug availability, patient preference and cancer activity (guidance).	
NICE 2020^a^ (73)		• Consider DOAC if active cancer and confirmed proximal DVT or PE. • If DOAC unsuitable, consider LMWH alone or VKA (following initial LMWH). • Choice of anticoagulant should consider tumor site, drug-drug interactions and bleeding risk.	• Review treatment at 3–6months according to clinical need.	
ACCP 2021^b^ (76)		• Apixaban, edoxaban or rivaroxaban (strong recommendation). ─ Apixaban or LMWH may be preferred in luminal GI malignancies.	• Extended-phase (> 3months) DOAC therapy (apixaban, edoxaban or rivaroxaban) (strong recommendation) Reassess periodically.	
ASH 2021^b^ (74)		• DOAC (apixaban or rivaroxaban) or LMWH (conditional recommendation). – Caution with DOACs in GI cancers.	• Treat for 3–6 months with a DOAC (apixaban, edoxaban or rivaroxaban) over LMWH or VKA (conditional recommendations). • Treat for > 6 months rather than short-term (3–6months) in patients with active cancer (conditional recommendation). Suggest continuing indefinitely rather than stopping after completion of a definitive period of anticoagulation (conditional recommendation). Use a DOAC or LMWH (conditional recommendation).	
NCCN 2022^b^ (75)	• Apixaban (category 1), edoxaban after ≥ 5days of parenteral anticoagulation (category 1) or rivaroxaban (category2A) preferred for patients without gastric or gastroesophageal lesions. Apixaban may be safer than edoxaban or rivaroxaban for patients with gastric or gastroesophageal lesions (category 2B) Caution in GU tract lesions. • LMWH (dalteparin) preferred for patients with gastric or gastroesophageal lesions (category1). • Dabigatran if above regimens not appropriate or unavailable.		• ≥ 3months or as long as active cancer or cancer therapy.	

ACCP, American College of Chest Physicians; ASCO, American Society of Clinical Oncology; ASH, American Society of Hematology; CrCl, creatinine clearance; ESC, European Society of Cardiology; GI, gastrointestinal; GU, genitourinary; ISTH, International Society on Thrombosis and Haemostasis; ITAC, International Initiative on Thrombosis and Cancer; LMWH, low molecular weight heparin; NCCN, National Comprehensive Cancer Network; NOAC, non-vitamin K oral anticoagulant; PE, pulmonary embolism; RCT, randomised controlled trial; UFH, unfractionated heparin; VKA, vitamin K antagonist ; VTE, venous thromboembolism

^a^Recommendations based on Hokusai VTE Cancer and SELECT-D trial results; ^b^Recommendations based on ADAM VTE, CARAVAGGIO, Hokusai VTE Cancer and SELECT-D trial results; ^c^High bleeding risk includes patients with luminal gastrointestinal cancers with an intact primary; cancers at risk of bleeding from the genitourinary tract, bladder, or nephrostomy tubes; or active GI mucosal abnormalities (e.g., duodenal ulcers, gastritis, esophagitis, or colitis)

Recommendations for the duration of anticoagulant treatment in patients with cancer (Table4) are mostly based on expert opinion, as high-quality data on extended treatment are limited [1, 36]. The over-arching principle is to continue anticoagulation if high risks for recurrence persist and in the absence of significant bleeding risks. Studies are ongoing to investigate extended treatment and include the 12-month EVE (NCT03080883) and API-CAT (NCT03692065) studies, which are comparing apixaban 5mg (standard dose used in CARAVAGGIO) versus stepped down 2.5mg dosing [36, 76]. In the metastatic disease setting, prescription of life-long anticoagulation is recommended by some experts, while others recommend standard 6 months of treatment with consideration of life-long anticoagulation after further discussion with the patient if there is evidence of VTE. The importance of individualized treatment regimens and shared decision-making in the management of patients requiring anticoagulation for CAT is emphasized in international guidelines [68].

While a number of Asian countries have national guidelines for the treatment of VTE, these guidelines generally incorporate recommendations from outdated ACCP guidelines, published in 2016 [77], and do not provide guidance for the use of DOACs to treat CAT [10, 28]. ASCO and ESC guidelines are also widely used in Asia [29, 30]. Unfortunately, these guidelines are not recent enough to include recommendations in line with the most recent RCT data on the treatment of CAT with DOACs [28, 30].

## Challenges in application of clinical guidelines

Although recently published guidelines incorporate recommendations for the use of DOACs in the treatment of CAT, many management questions lack clinical trial data to inform evidence-based recommendations [78]. Clinical challenges for which empiric management decisions must be made include the presence of GI or GU tumors (with intact primary tumor), concomitant anticancer therapies (particularly antiangiogenic therapies), incidentally discovered VTE, extremes of body weight, thrombocytopenia, and renal impairment [61, 79].

While the optimal duration of therapy is uncertain, it is generally accepted that anticoagulation should be continued in patients with active cancer or in patients with cancer in remission, who are still receiving chemotherapy with associated thrombotic risk, unless there are contraindications or an unacceptable clinical risk [68–71, 74]. Risk assessment scores could potentially be used to identify patients who may derive the most benefit from extended anticoagulation (≥ 6 months after the index event), but the Ottawa score, which is the only risk assessment score available to predict recurrent VTE in cancer patients [80, 81], has not been validated in Asian patient cohorts [11]. Compared with the US population, a higher rate of GI cancers and predominance of VKA therapy may have contributed to lack of relevance of the Ottawa score when applied to a cohort of Korean patients with CAT [82].

Of particular note in Eastern Asia, where the incidence and mortality of gastric cancer is the highest worldwide [83], caution is advised with edoxaban and rivaroxaban in patients with GI cancer because of an increased risk of major GI bleeding versus dalteparin in the Hokusai VTE Cancer and SELECT-D trials [49, 50]. However, the absence of an increased risk of major upper or lower GI bleeding with apixaban versus dalteparin in the CARAVAGGIO trial, including in patients with GI malignancies, suggests that apixaban could be a safe alternative to LMWH in these patients [51, 55, 84]. There have been suggestions of ethnic differences in the risk of GI bleeding in patients treated with DOACs versus LMWH. While the risk of GI bleeding was not significantly different between DOACs and LMWH in the Hokusai VTE cancer and SELECT D studies that were predominated by non-Asian patients, the real-world study in Taiwanese patients showed significantly lower risk of major GI bleeding with DOACs compared with LMWH [18]. This finding supports GI safety of DOACs in Asians. Nevertheless, larger population-based studies are required to validate this observation.

Asians are known to have lower body mass index than non-Asian populations [10], and clinicians may have concerns about using full-dose DOACs in patients with low body weight (< 60kg). Whereas half-dose edoxaban was used in patients with body weight ≤ 60kg in the Hokusai VTE Cancer trial [49], there is no strong evidence for DOAC dose reduction in low-weight patients. Based on SELECT-D, ADAM VTE and Caravaggio [49–51], in which all patients received full-dose rivaroxaban or apixaban, clinicians should feel comfortable using standard doses of these DOACs regardless of body weight [74].

In the setting of thrombocytopenia, which is a common consequence of chemotherapy and/or malignancy, the risk of bleeding is increased but the risk of VTE remains [84]. Patients with acute leukemia were excluded from CARAVAGGIO because of increased risk of bleeding associated with low platelet counts [51]. Furthermore, all four RCTs comparing DOACs with dalteparin in patients with CAT excluded patients with severe thrombocytopenia (Hokusai VTE Cancer and ADAM VTE trials: platelet count < 50 × 10^9^/L; SELECT-D trial: <100 × 10^9^/L; Caravaggio trial: <75 × 10^9^/L) [49–52]. For patients with platelet count < 25 × 10^9^/L, it is safest to withhold anticoagulation until the platelet count has recovered [84]. Analysis of medical records in Taiwan showed that patients with platelet counts < 50 × 10^9^/L treated with DOACs had a higher risk of recurrent VTE, composite VTE, and major bleeding than patients treated with LMWH [18]. In such patients, dose-adjusted LMWH may be preferred but a half-dose DOAC has been suggested as a potentially acceptable alternative [84]. Care should be taken when using DOACs in patients with an expected decrease in platelet count during chemotherapy [67].

Patients’ needs and preferences must be considered and reviewed when choosing and receiving the appropriate anticoagulant, keeping in mind the risk of bleeding, tumor type, renal function, drug–drug interactions, and financial costs [1, 54, 61, 79, 85]. For patients undergoing treatment for active cancer, ASCO recommends that oncologists and members of the oncology team educate patients regarding VTE, particularly in settings that increase risk, such as major surgery, hospitalization, and while receiving systemic antineoplastic therapy [70]. While DOACs have been approved for the treatment of VTE in many Asian countries, only a few countries provide reimbursements to patients [29].

## Summary and conclusions

DOACs are a convenient, effective and safe option for many patients and represent a major paradigm shift in the treatment of CAT worldwide [54, 86]. Although Asian patients with cancer were rarely included in pivotal RCTs of DOACs for the treatment of CAT [49–52], data from general VTE RCTs indicate that DOACs have similar efficacy to standard of care treatment with no increased safety concerns in Asian populations [10]. Although RCTs specifically addressing the efficacy and safety of DOACs in Asian patients with CAT are warranted, real-world clinical evidence supports factor Xa inhibitor DOACs as an effective and safe alternative to LMWH for the treatment of CAT in Asian patients [18, 28, 63]. Appropriate utilization of these convenient agents is especially important for patients with cancer who already carry a major burden of disease. The most recently updated international guidelines recommend the factor Xa inhibitors apixaban, edoxaban or rivaroxaban as first-line therapy for CAT in patients without GI or GU cancers at low risk of bleeding, and who have no potential for drug–drug interactions [73–75]. In other patients, current practice favors LMWH, although it is now acknowledged apixaban may be safer than edoxaban and rivaroxaban, and an alternative to LMWH in patients with GI malignancies [74, 75]; VKAs are not recommended and should be reserved for patients for whom LMWHS/DOACs are unavailable or unsuitable. The findings of the CARAVAGGIO trial support the use of apixaban in GI cancer and therefore expand the proportion of patients with CAT who are potentially suitable for treatment with DOACs [51, 55]. Although it remains unclear whether apixaban is safer than edoxaban or rivaroxaban in patients with GI cancer, it is possible that compared with once daily edoxaban or rivaroxaban dosing, more stable plasma drug concentrations, with lower peaks and higher troughs, obtained with twice-daily apixaban dosing could have an impact on safety [87, 88]. Caution is, however, still advised, particularly in patients with upper GI tumors or unresected lower GI tumors. In all such patients, the decision to use any DOAC requires careful consideration of bleeding risk, the cost-benefit and convenience of oral therapy, and patient needs and preferences, which may change over the course of the cancer journey.

While recommendations made here are based on the currently available data, this review calls for randomized trials designed specifically to evaluate the efficacy and safety of DOACs in the prophylaxis and treatment of CAT in Asian patients. These trials should address the optimal dosage and duration of therapy to maximize the benefits and minimize the risks of anticoagulation in patients with different types of cancers.

## Data Availability

Not applicable.

## Declarations

Gwo Fung Ho has received payment or honoraria for lectures, presentations, speakers’ bureaus, manuscript writing or educational events for Boehringer Ingelheim, Pfizer, and AstraZeneca; support for attending meetings and/or travel from AstraZeneca and Bristol Myers Squibb; and has had a leadership or fiduciary role in other board, society, committee or advocacy group, paid or unpaid, for Boehringer Ingelheim, Pfizer, AstraZeneca, Eli Lily, and Merck Sharp & Dohme. Gwo Fung Ho’s affiliated institution has received grants or contracts from AstraZeneca, Regeneron, Astellas, Merck Sharp & Dohme, Pfizer, Arcus Biosciences, Roche, and Adipolab.

Rajiv Parakh has received honoraria for presentations and speaker bureau for Bayer, Boehringer Ingelheim, and Pfizer; and been a member on advisory boards for novel oral anticoagulants for Daitchi Sankyo, Bayer, Boehringer Ingelheim, and Pfizer.

Nannette Rey has received honoraria for speakers bureaus for Boehringer Ingelheim, Servier, and Sanofi; has participated on an advisory board for Boehringer; and was the President of the Philippine Heart Association 2018–2019.

Alexander Cohen has received contracts and consulting fees for other projects from Bristol Myers Squibb and Pfizer; payments for lectures and presentations from Bristol Myers Squibb and Pfizer; and Advisory Board fees for other projects from Bristol Myers Squibb and Pfizer, all paid to a consulting company.

Lai Heng Lee, Pongwut Danchaivijitr, Noppacharn Uaprasert, Harinder Gill, Paresh Pai, and Jen-Kuang Lee have nothing to declare.

## Abbreviations

ACCP: American College of Chest Physicians.
ASCO: American Society of Oncology.
ASH: American Society of Hematology.
CAT: cancer-associated thrombosis.
CI: confidence interval.
CRNMB: clinically relevant non major bleeding.
DOAC: direct oral anticoagulant.
ESC: European Society of Cardiology.
GI: gastrointestinal.
GU: genitourinary.
HR: hazard ratio.
INR: international normalized ratio.
ISTH: International Society on Thrombosis and Haemostasis.
ITAC: International Initiative on Thrombosis and Cancer.
LMWH: low molecular weight heparin.
NCCN: National Comprehensive Cancer Network.
NICE: National Institute for Health and Care Excellence.
RCT: randomized controlled trial.
VKA: vitamin K antagonist.
VTE: venous thromboembolism.
